# Negative Pressure Pulmonary Edema after Laryngospasm: A Revisit with a Case Report

**DOI:** 10.4172/2155-6148.1000252

**Published:** 2013-10-28

**Authors:** Lourdes Al Ghofaily, Courtney Simmons, Linda Chen, Renyu Liu

**Affiliations:** Department of Anesthesiology and Critical Care, Perelman School of Medicine at the University of Pennsylvania, USA

## Introduction

Laryngospasm, a brief closure of the vocal cords is not an uncommon perioperative occurrence. If recognized and managed appropriately, the effects are transient and reversible. However, in rare cases where recognition and management are delayed, the consequences are associated with a high morbidity including desaturation, awareness, negative pressure pulmonary edema, and mortality. This case highlights that of a healthy woman admitted the intensive care unit (ICU) for negative pressure pulmonary edema (NPPE) after an episode of laryngospasm.

## Case Presentation

A 22 year-old woman (ASA-PS II, ht 167.6cm, wt 63.0kg, BMI 22) with a strong family history of breast and ovarian cancer status post prophylactic bilateral mastectomies and one-stage reconstruction presented for implant removal and capsulotomy under general anesthesia. Further medical history included sickle cell trait and alpha thalassemia trait. Surgical history included cesarean section under epidural anesthesia. Upon examination of the airway, she was a Mallampati I with good oral opening, normal thyromental distance, and full neck range of motion. On the day of surgery, the patient was afebrile with a non-productive cough thought to be non-infectious in etiology.

After receiving intravenous midazolam 2 mg, she underwent a smooth, routine intravenous induction with lidocaine 100 mg and propofol 200 mg with an uneventful placement of a 3.5 laryngeal mask airway (LMA) (AirQ, United States, St. Louis, Missouri 63117-1427). She was maintained on end-tidal sevoflurane 1.75%–2.35% and received morphine 12 mg. Surgery was uneventful and upon emergence from anesthesia, the patient had sudden onset of laryngospasm before LMA removal. This was recognized due to inability to ventilate through the LMA and decreasing oxygen saturation. The LMA could not be removed as the patient was biting down and was not following commands. She was given positive pressure via LMA without improvement and ultimately received intravenous succinylcholine 60 mg. She experienced a transient desaturation to 65% and quickly recovered to 100% as manual ventilation through the LMA gradually became easier. She was taken to the post-anesthesia care unit (PACU) on 6L oxygen via nasal cannula without further events.

**Figure 1:**
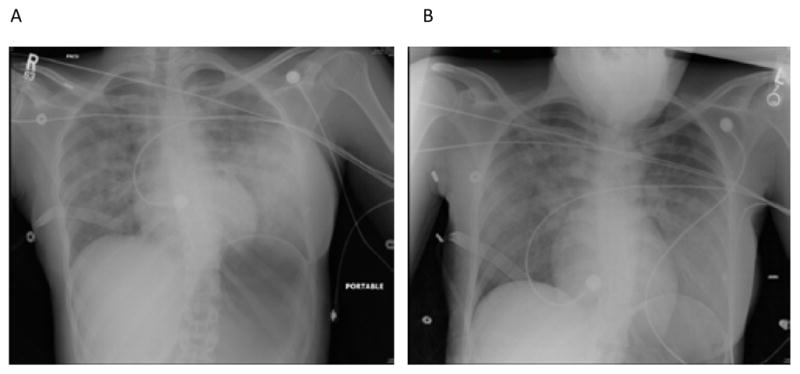
Postoperative chest x-rays. Figure 1A is the chest X-ray performed 1 hour after surgery in the PACU. Moderate pulmonary edema is demonstrated. Figure 1B is the chest X-ray obtained in the morning of postoperative day 1. Improving pulmonary edema, likely hydrostatic, is demonstrated.

Her PACU course was significant for a persistent productive cough with white mucous and she was placed on 100% FIO_2_ non-rebreather for comfort. A chest X-ray (Figure 1A) revealed moderate pulmonary edema. She was admitted to the surgical intensive care unit (SICU) overnight for monitoring with the presumed diagnosis of negative pressure pulmonary edema and was given one dose of intravenous furosemide 20 mg. She was weaned to room air overnight. On post-operative day (POD) 1, her chest X-ray showed mild improvement (Figure 1B). She was advanced to a regular diet and was transferred to the floor. She was discharged home on POD 2.

## Discussion

Laryngospasm is a well-known and feared complication of LMA usage. Several factors may predispose patients to laryngospasm including recent upper respiratory tract infections, male gender, young age, dry cough, and history of reactive airway disease [1]. Laryngospasm results from a reflex arc. The trigeminal, glossopharyngeal, and vagus (via superior and inferior laryngeal nerves) provide the afferent pathways that innervate the mucosal surfaces of the nasopharynx to the vocal cords. Stimuli include secretions, blood, gastric fluid, pressure, and temperature changes. Smooth and skeletal muscle stimulation can result in coughing, bronchospasm, apnea, and vocal cord closure. In the anesthetized or lightly anesthetized patient, the vocal cord closure may be prolonged due to dysregulation from higher centers and thus can result in apnea, stridor, coughing, and clinically evident desaturation. Blood pressure and heart rate fluctuations are mediated first through the vagus nerve and result in bradycardia. Continued laryngospasm eventually results in hypoxia. At this point, the sympathetic stimulation overrides the parasympathetic innervations resulting in tachycardia and hypertension. Severe hypoxemia will eventually results in severe bradycardia and asystolic arrest. Bradycardia is more common in children [1,2].

Visvanathan, et al. [2] examined 187 cases of laryngospasm among 4000 patients. 61% experienced significant desaturations, 35% of this study group had major physiological changes, 3% suffered pulmonary edema, 3% suffered aspiration, and 1% suffered cardiac arrest.

Proper treatment involves administering 100% oxygen, ceasing stimulation to the patient, clearing the airway secretions, and administering positive pressure. If this does not resolve the laryngospasm, an intravenous induction agent should be given to deepen anesthesia. If this still does not resolve, intravenous succinylcholine 0.5 mg/kg should be given when there are no contraindications. In the event of no intravenous access, intramuscular succinylcholine 4 mg/kg is also a treatment option. If there is a contraindication to succinylcholine, intravenous atropine 0.01 mg/kg is the next best choice since the efferent arc has substantial innervation through the vagus nerve [2,3]. Of note, laryngospasm in children is accompanied by significant bradycardia, and atropine should be considered earlier for treatment in this group of patients [2,3]. If all these measures fail, intubation is indicated [2,3].

Pulmonary edema and aspiration should be ruled out after episode of laryngospasm needing muscle relaxant. A chest radiograph is needed for differential diagnosis. This patient’s post-operative chest X-ray obtained in the PACU revealed moderate pulmonary edema as well as a significant gastric bubble, likely from vigorous positive pressure ventilation when the vocal cords were closed. A gastric tube might be helpful to decompress the stomach to prevent decreased diaphragmatic excursion [12]. However, the passage of a gastric tube could further stimulate the oropharynx and upper larynx and precipitate a second episode of laryngospasm.

Despite the lack of post-operative hypoxia with this episode of laryngospasm, the patient was admitted to the SICU with a presumptive diagnosis of negative pressure pulmonary edema. NPPE is a relatively rare but potentially dangerous form of non-cardiac pulmonary edema that results from vigorous patient inspiration against an obstructed upper airway. It is reported that the prevalence for NPPE is less than 0.1% [4]. However, this figure could be grossly underestimated due to the clinical picture of NPPE that is often associated with other pulmonary processes, for example cardiogenic pulmonary edema, ARDS, or multifocal pneumonia. As reported in this patient, most post-obstructive pulmonary edema patients are young and healthy [5].

As with any anesthetic complication, disclosure of the event to the patient should occur. Follow-up with this patient occurred in the ICU. At that time, she denied recollection of the event. Confirmation that patients do not have awareness during these episodes should always be sought [3,9].

While a consensus has been reached in the literature regarding the profile of the patient population of NPPE, the pathogenesis of this pulmonary disorder is still heavily debated. It is widely accepted that the cause is multi-factorial with a central mechanism involving the generation of a large inspiratory force against an obstructed upper airway, which triggers an accumulation of high negative intra-pleural pressure in the range of −50 to −100 cm H_2_O. This high negative intra-pleural pressure creates a pressure gradient allowing for the extravasation of fluid from the pulmonary capillaries into the interstitial and alveolar spaces, and generates a large increase in venous return resulting in increased preload [4,6]. Concomitantly, hypoxia and sympathetic stimulation from laryngospasm increase mean arterial pressure and afterload causing decreased forward stroke volume.

Once relief of obstruction has taken place, evidence of NPPE can occur anywhere from minutes to hours later [4]. Signs of respiratory distress such as cough and tachypnea will be present along with a decrease in oxygen saturation and the inability to maintain oxygen saturation above 95% [6]. The hallmark sign of NPPE is pink, frothy sputum. Physical examination of the patient may reveal rales, coarse rhonchi, and occasional wheezes upon auscultation of the lung fields and diaphoresis. Chest radiograph findings can be positive for diffuse bilateral interstitial and alveolar infiltrates appearing as “whited out” areas [7]. Arterial blood gas samples can be normal or show an acute respiratory acidosis. Hemodynamic monitoring reveals normal right and left ventricular function, CVP, and pulmonary artery occlusion pressure [6]. Due to this relatively nonspecific clinical picture, a differential diagnosis must be carefully considered prior to diagnosing the patient with NPPE. Differential diagnoses include pulmonary aspiration; acute respiratory distress syndrome, pulmonary embolization, and intravascular volume overload [7].

Upon confirmation of a diagnosis of post-obstructive pulmonary edema, treatment is focused on improving respiratory function and avoiding further damage to the lung. This includes maintaining the airway and providing supplemental oxygen. Prolonged mechanical ventilation may be required. When conservative therapy yields little improvement, some suggest the use of intravenous diuretics despite no conclusive evidence of clinical benefits [4].

The best treatment for laryngospasm is prevention. The astute anesthesiologist is aware of the predisposing conditions that may place their patient at risk for laryngospasm and takes actions to avoid this situation. While formulating an anesthetic plan for a patient with a history of laryngospasm, securing the airway with an endotracheal tube can be considered, however, it is not completely protective of post-extubation laryngospasm [13]. Should the situation arise, rapid recognition and treatment will minimize any further associated risks. A clear understanding of the reflexes involved and their dysregulation aid in avoidance and treatment of this potentially fatal occurrence.
